# Detection of Coalescent Acute Mastoiditis on MRI in Comparison with CT

**DOI:** 10.1007/s00062-020-00931-0

**Published:** 2020-07-21

**Authors:** R. Saat, G. Kurdo, A. Laulajainen-Hongisto, A. Markkola, J. Jero

**Affiliations:** 1 HUS Medical Imaging Center, Radiology, University of Helsinki and Helsinki University Hospital, POB 340 Haartmaninkatu 4, HUS 00029 Helsinki, Finland; 2 Radiology, East Tallinn Central Hospital, Ravi tn. 18, 10138 Tallinn, Estonia; 3 Otorhinolaryngology and Head and Neck Surgery, University of Helsinki and Helsinki University Hospital, POB 263 Kasarmikatu 11–13, HUS 00029 Helsinki, Finland; 4 Otorhinolaryngology and Head and Neck Surgery, University of Turku and Turku University Hospital, POB 52 Kiinamyllynkatu 4–8, 20521 Turku, Finland

**Keywords:** Temporal bone, Imaging, Middle ear, Otitis media, Infection

## Abstract

**Purpose:**

Current imaging standard for acute mastoiditis (AM) is contrast-enhanced computed tomography (CT), revealing inflammation-induced bone destruction, whereas magnetic resonance imaging (MRI) outperforms CT in detecting intracranial infection. Our aim was to compare the diagnostic performance of MRI with CT in detecting coalescent AM and see to which extent MRI alone would suffice to diagnose or rule out this condition.

**Methods:**

The MR images of 32 patients with AM were retrospectively analyzed. Bone destruction was evaluated from T2 turbo spin echo (TSE) and T1 Gd magnetization-prepared rapid acquisition with gradient echo (MPRAGE) images. Intramastoid enhancement and diffusion restriction were evaluated subjectively and intramastoid apparent diffusion coefficient (ADC) values were measured. The MRI findings were compared with contrast-enhanced CT findings of the same patients within 48 h of the MR scan.

**Results:**

Depending on the anatomical subsite, MRI detected definite bone defects with a sensitivity of 100% and a specificity of 54–82%. Exception was the inner cortical table where sensitivity was only 14% and specificity was 76%. Sensitivity for general coalescent mastoiditis remained 100% due to multiple coexisting lesions. The absence of intense enhancement and non-restricted diffusion had a high negative predictive value for coalescent mastoiditis: an intramastoid ADC above 1.2 × 10^−3^ mm^2^/s excluded coalescent mastoiditis with a negative predictive value of 92%.

**Conclusion:**

The MRI did not miss coalescent mastoiditis but was inferior to CT in direct estimation of bone defects. When enhancement and diffusion characteristics are also considered, MRI enables dividing patients into low, intermediate and high-risk categories with respect to coalescent mastoiditis, where only the intermediate risk group is likely to benefit from additional CT.

## Introduction

Despite the decreased incidence of acute mastoiditis (AM) in the antibiotic era, AM cases still occur and require prompt and effective treatment to avoid life-threatening complications [1, 2]. Although diagnostic and treatment algorithms rely mostly on the clinical picture, imaging is required when complications such as coalescent AM (AM with inflammatory bone destruction) or intracranial spread are suspected. Imaging may also support clinical decisions in equivocal situations, such as to estimate the need for surgery when the response to conservative treatment is suboptimal [3, 4]. Some authors recommend imaging all AM patients to exclude clinically silent complications [5–7].

The current standard for imaging AM is contrast-enhanced high-resolution CT, due to its ability to detect inflammation-induced bone destruction [8, 9]. In detecting intracranial infection, however, MRI has shown higher sensitivity than CT [10–15]. Therefore, some patients undergo both CT and MRI scans.

Recent studies have evaluated the use of MRI not only for showing intracranial complications of AM (epidural and subdural abscess, pachymeningitis and leptomeningitis, dural venous sinus thrombosis) but also for identifying signs of infection inside the temporal bone itself, including bone destruction [17–19]. In the context of AM, bone usually becomes visible on MRI due to the intramastoid mucosal swelling and inflammatory secretions that replace the air and provide highly intense background for the signal-void bony structures on T2-weighted images [17]. Additionally, contrast enhancement and diffusion restriction of the intramastoid contents are likely to correlate with the degree of mastoid inflammation.

Our primary aim was to evaluate the diagnostic performance of MRI in detecting coalescent AM in comparison with CT. If MRI were able to detect coalescent AM, CT could possibly be omitted in the future when a patient has already undergone an MRI scan to exclude intracranial complications.

## Material and Methods

### Design

This study was conducted retrospectively at an academic tertiary hospital with a referral population of approximately 1.6 million. Study permission was obtained from the institutional ethics committee with a waiver of informed consent.

### Study Population

All patients hospitalized at the otorhinolaryngology department between 2003 and 2016 due to AM infection (International Classification of Diseases 2010 code H70.0) with both contrast-enhanced CT and MRI scans of temporal bones from the same day or consecutive days during hospitalization were enrolled in the study. Exclusion criteria were the following: ipsilateral mastoidectomy prior to either of the imaging studies, AM in a chronically infected ear with cholesteatoma, underlying temporal bone tumor and middle ear sarcoidosis proven by intraoperative biopsy. The final study group had 32 patients.

### Clinical Data

Clinical data regarding patient age, gender, time of AM diagnosis and imaging studies, clinical symptoms and treatment were collected retrospectively from electronic patient records. All diagnoses were re-validated by a dedicated otorhinolaryngologist according to previously described criteria [20–22] and categorized into classical AM (symptom duration ≤14 days) or latent AM (symptoms persisting >14 days) in order to more specifically describe our patient cohort.

### Image Acquisition and Evaluation

The CT scans were performed with a preset 50 s delay after intravenous injection of 1.5 ml/kg iohexol 350 mg/ml contrast agent at 3 ml/s injection rate on various 16-row or 64-row multidetector CT scanners. Scans were acquired with straight gantry, a 120 kV tube voltage and tube current of 90–150 mA or quality reference 220mA. Images were reconstructed in the axial plane of lateral semicircular canals and the coronal plane perpendicular to it, with bone and soft tissue kernels and a section thickness of 0.4–0.6 mm (30 patients) or 1 mm (2 patients).

The MRI scans were performed on various 1.5 T scanners in 30 patients and on a 3 T scanner in 2 patients with head and neck coils. The standard protocol consisted of axial and coronal T2 turbo spin echo (TSE) and axial T1 spin echo; axial echo-planar diffusion-weighted imaging (EPI DWI) and apparent diffusion coefficient (ADC); axial isotropic T2-weighted constructive interference in steady state (CISS); and isotropic T1 magnetization-prepared rapid acquisition with gradient echo (MPRAGE) images with intravenous gadoterate meglumine (Dotarem; Guerbet, Aulnay-sous-Bois, France) 0.1 mmol/kg, obtained in the sagittal plane and reconstructed in axial and coronal planes parallel to and perpendicular to the anterior skull base. Details of the protocol are shown in Table 1. Duration of the whole protocol was 30 min.

**Table 1:** Details of the MRI protocol

Sequence	Thickness/gap (mm)	Matrix	FOV (mm)	TR/TE (ms/ms)	Flip angle (°)
T2 TSE tra	3/0.3	384	230	5000/86	150
T2 TSE cor	3/0.3	384	170	4760/82	150
T1 SE tra	3/0.3	256	200	450/8.7	90
EPI DWI^a^/ADC tra	4/0.6	192	230	3000/89	90
CISS tra	0.7/0	512	200	11.56/5.78	80
3D T1 MPRAGE Gd^b^	1/0	256	260	1900/3.09	15

*T2 TSE* T2 turbo spin echo, *T1 SE* T1 spin echo, *EPI DWI/ADC* echo-planar diffusion-weighted imaging, *CISS* constructive interference in steady state, *3D T1 MPRAGE* Gd 3‑dimensional isotropic T1 magnetization-prepared rapid acquisition with gradient echo with intravenous gadoterate meglumine, *tra* transaxial plane, *cor* coronal plane

^a^b factor = 0 and 1000 s/mm^2^

^b^Acquisition in sagittal plane, reconstructions in axial and coronal plane

The anonymized CT and MRI scans were independently assessed from picture archiving and communication system (PACS) by a board-certified neuroradiologist and a board-certified head and neck radiologist with 7 and 8 years of subspecialty working experience, respectively, during reading sessions several months apart. Discrepancies between opinions were resolved by additional joint reading sessions, separately for CT and MRI scans and CT was the reference standard for MRI findings.

**Fig. 1:**
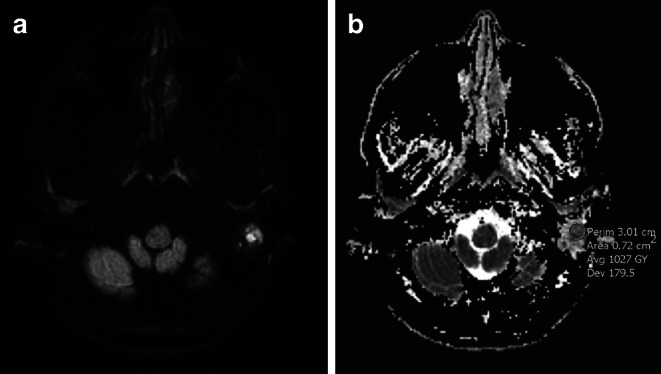
Restricted intramastoid diffusion due to purulent infection on axial DWI trace map (**a**) and ADC map (**b**) of the skull base. Note that mastoid may simultaneously include areas with different diffusion restriction. Measurement of ADC values was performed from areas with the most restriction, such as on **b**

On MRI, bone destruction was estimated as lack of definition of bony septa or cortical bone on T2 TSE and T1 Gd MPRAGE images and classified into the following categories: (1) no destruction or (2) suspected or definite destruction. In only partially fluid-filled mastoids septa are not directly assessable on MRI and in these cases septa were assumed to be intact. Additionally, intense mastoid enhancement (approaching the signal intensity of the sigmoid sinus on T1 Gd MPRAGE) and intramastoid diffusion restriction (defined as signal intensity hyperintense to cerebellar parenchyma in DWI, b = 1000 and a signal drop in ADC) were recorded as previously described [17]. For ADC measurement, three different regions of interest (ROI) were placed, each in a different section, on an area with the most diffusion restriction within the mastoid, avoiding the cortical bone and aerated regions and areas with visible artifacts (Fig. 1). Mean values were calculated for each patient.

**Fig. 2:**
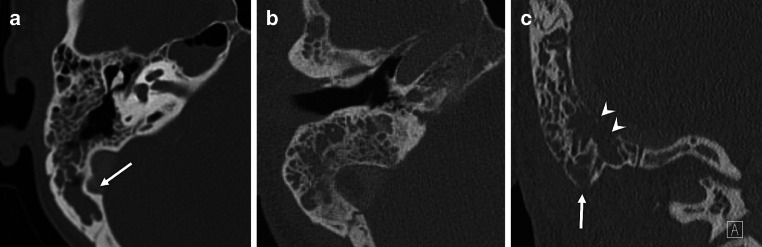
Bone destruction on CT axial (**a**, **b**) and coronal planes (**c**) of the right temporal bone: **a** no bone destruction (*arrow* marks an emissary vein), **b** demineralization, **c** definite bone destruction (*arrow* marks an external cortical defect at the tip of the mastoid and *arrowheads* mark an internal cortical defect towards the sigmoid sinus)

In CT, bone destruction was estimated from images reconstructed with the bone kernel separately at three anatomical subsites: the mastoid septa, the inner cortical table and the outer cortical table. Bone destruction was divided into the following categories: (1) no destruction, (2) demineralization or suspicious destruction or (3) definite destruction (Fig. 2). Mastoiditis was called coalescent if definite bone destruction at one or more of the subsites was present on CT.

Complications were recorded both in CT and MRI according to a following structure: perisinuous lesions; epidural abscess outside the perisinuous region; venous sinus thrombosis outside the perisinuous region; extracranial abscess; pachymeningitis or leptomeningitis; and other intracranial complications, if any. Perisinuous lesions (contrast filling defects between the sigmoid sinus and the mastoid) were divided into the following four categories as described previously [23]: grade I: no halo, grade II: linear halo, grade III: nodular halo of <4 mm thickness and grade IV: nodular halo of ≥4 mm thickness (the last two categories likely referring to a mural thrombus or an epidural abscess at the sigmoid sinus wall). Other imaging findings were simply recorded as present or absent.

### Statistical Analysis

The Cohen kappa or kappa with linear weighting score (when appropriate) were calculated to estimate agreement of opinions between the two radiologists. Associations between categorical CT and MRI findings were determined with two-sided Fisher’s exact test. Comparison of mean ADC values between the groups with and without bone destruction was performed by Mann-Whitney U‑test. Sensitivities, specificities, and positive and negative predictive values were calculated for categorical MRI parameters in comparison with CT. Statistical analysis was performed with IBM SPSS Statistics for Windows, Version 24.0. (IBM Corp, Armonk, NY, USA). A *p*-value of <0.05 was considered significant.

Additionally, a receiver operating characteristic (ROC) analysis with multiple logistic regression was conducted to calculate the area under the curve (AUC) for ROC to determine the optimal ADC threshold for predicting bone destruction in AM, along with corresponding accuracy measurements.

## Results

### Patients

The final study group consisted of 32 pediatric and adult patients (13 male, 19 female) with consecutive contrast-enhanced CT (CECT) and MRI scans. The mean age was 33 years (median 35 years, range 4–81 years) and 9 patients (28%) were children. Anesthesia was required for one MRI scan of a 4-year-old child but the rest of the children (age range 5–14 years) were scanned without anesthesia. Infection was on the left in 14 (44%) and on the right side in 18 (56%) patients. The mean time from AM diagnosis to MRI was 0.53 days (median 0, range 0–5 days). The mean time from CT to MRI was 0.03 days (median 0; range −1 to 1 days). Classical AM was present in 24 (75%) and latent AM in 8 (25%) patients. Mastoidectomy was performed on 20 (63%) patients.

### Imaging Findings

**Fig. 3:**
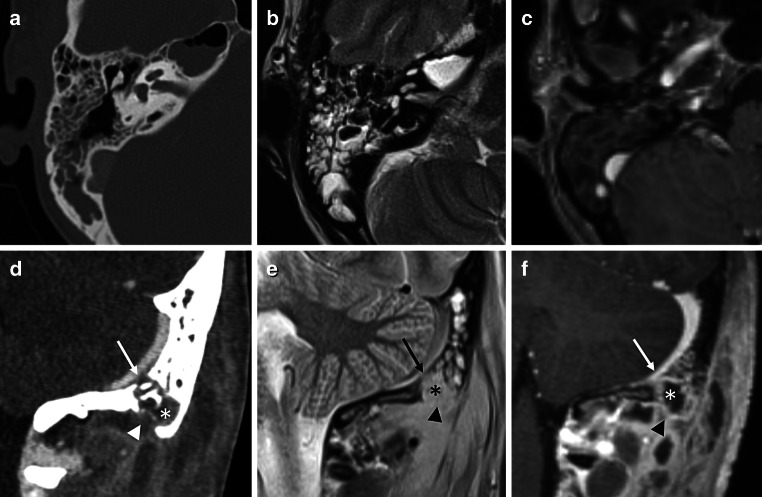
Images of the right temporal bone in CT (**a**), MRI, T2 TSE (**b**), and MRI, T1 Gd MPRAGE (**c**) showing no bone destruction. Images of the left temporal bone in  CT (**d**), MRI T2 TSE (**e**), and MRI T1 Gd MPRAGE (**f**) showing bone destruction of septa (*asterisk*), inner cortical table (*arrow*) and outer cortical table (*arrowhead*) of the mastoid

The prevalence rates for bone destruction at different anatomical subsites (Fig. 3) with corresponding kappa values in CT and MRI are presented in Table 2.

**Table 2:** Bone destruction at different anatomical subsites in CT and MRI: prevalence and interobserver agreement

Anatomical subsites	CT		MRI	
	N^a^ ^+^ ^b^ (%)	Kappa	*n* (%)	Kappa
*Mastoid septa*	7 + 5 (22 + 16)	0.62	11 (34)	0.28
*Inner cortical table*	3 + 7 (9 + 22)	0.69	7 (22)	0.08
*Outer cortical table*	4 + 4 (13 + 13)	0.73	9 (28)	0.74
*Bone destruction in general*	7 + 9 (22 + 28)	0.70	18 (56)	0.43

*CT *computed tomography, *MRI* magnetic resonance imaging

*n*^*a*^ ^*+*^ ^*b*^ the number of bone destructions in CT is given separately for suspicious^a^ and definite^b^ lesions

The sensitivity, specificity and positive (PPV) and negative predictive values (NPV) in detecting definite bone lesions on MRI (T2 TSE or T1 Gd MPRAGE) were as follows: mastoid septa (100%, 78%, 46%, 100%), outer cortical table (100%, 82%, 44%, 100%), inner cortical table (14%, 76%, 14%, 76%) and coalescent mastoiditis in general (100%, 54%, 42%, 100%).

Intense mastoid enhancement (kappa = 0.69) was present in 17 (53%) patients and diffusion restriction in 21 out of 29 (72%) patients. Both had low specificity when predicting definite bone destruction (intense mastoid enhancement 52–57%; diffusion restriction 32–38%, depending on the anatomical subsite); however, the sensitivity and NPV were 100% for DWI in predicting definite bone destructions regardless of anatomical subsites. For intense mastoid enhancement, the sensitivity and NPV were 100% in predicting septal and outer cortical destructions. Mastoid enhancement was less sensitive in predicting IC destruction (sensitivity 71%; NPV 87%) and accordingly, bone destruction in general (sensitivity 78%; NPV 87%).

The ADC values from the obliterated mastoid ranged from 0.64 to 1.70 × 10^−3^ mm^2^/s. The mean ADC values differed in groups with (0.93, 95% confidence interval, CI 0.61–1.25 × 10^−3^ mm^2^/s) and without (1.29, 95% CI 1.19–1.39 × 10^−3^ mm^2^/s) definite septal destruction and with (0.86, 95% CI 0.48–1.24 × 10^−3^ mm^2^/s) and without (1.29, 95% CI 1.20–1.38 × 10^−3^ mm^2^/s) definite outer cortical destruction. Differences were not significant regarding the inner cortical destruction (1.07 versus 1.27 × 10^−3^ mm^2^/s) and coalescent mastoiditis in general (1.05 versus 1.30 × 10^−3^ mm^2^/s, *p* = 0.067).

The optimal ADC cut-off threshold for coalescent mastoiditis was 1.174 × 10^−3^ mm^2^/s (AUC ROC = 0.72). An ADC value of ≤1.2 × 10^−3^ mm^2^/s had 86% sensitivity, 71% specificity and 44% PPV for coalescent mastoiditis. An ADC value higher than 1.2 × 10^−3^ mm^2^/s had 92% NPV for coalescent AM. The ADC values were available from 29 patients; 25 were scanned with the same MRI unit. Discarding the remaining four patients from ROC analysis did not change the threshold values.

#### Complications

The number of detected extracranial abscesses was four (13%) in CT (kappa = 1) and six (19%) in MRI (kappa = 0.90).

Positive perisinuous findings were detected in two (grade II), four (grade III) and four (grade IV) patients in CT (kappa = 0.84) and in one (grade II), two (grade III) and four (grade IV) patients in MRI (kappa = 0.86). Of the ten patients with inner cortical defects, five had grade III or IV perisinuous lesions, consistent with thrombus or abscess. Additionally, there were three patients with grade III or IV lesions but without underlying bone defects.

Outside the perisinuous area, one epidural abscess (3%) and one venous sinus thrombosis (3%) was detected in the same patients both by CT and MRI. Additionally, MRI alone detected three cases of pachymeningitis (9%), two of leptomeningitis (6%) and one temporal lobe cerebritis. Of these, inner cortical defect was only present in one patient having simultaneously pachymeningitis and leptomeningitis and temporal lobe cerebritis.

## Discussion

The study compared MRI with CT in detecting coalescent AM.

The MRI was 100% sensitive in detecting osteolytic lesions of mastoid septa and external cortical table from T2 TSE or T1 Gd MPRAGE images. The sensitivity in detecting definite internal cortical defects was low (14%). Despite this coalescent mastoiditis in general was not missed on MRI due to multiple coexisting bone lesions at different anatomical subsites in coalescent AM. The specificity of T2 TSE and T1 Gd MPRAGE for identifying definite bone destruction was moderate, ranging from 76% to 82% at different anatomical subsites and being 54% for coalescent mastoiditis in general. The interobserver agreement was also lower than in CT. Substantial agreement, similar to CT, was only achieved with respect to outer cortical lesions.

Both intense intramastoid enhancement and diffusion restriction were frequent signs in AM. Occurring more often than bone destruction, they were not useful to predict coalescence. Their absence, however, was useful for ruling out coalescent mastoiditis (NPV of 100% for non-restricted diffusion and 87% for non-intense enhancement). Enhancement sensitivity for bone defects also remained as the least sensitive at the inner cortical table. Nor did the ADC values differ statistically among patients with or without inner cortical defects, although there was a difference in ADC values among patients with or without septal and outer cortical destruction. The ADC values higher than 1.2 × 10^−3^ mm^2^/s had a 92% NPV for coalescent AM. It must be kept in mind, when reading the temporal bone MRI, that other entities within the temporal bone or in its vicinity, most notably a cholesteatoma, may have an intense signal on DWI [24]. Using the ADC maps, paying close attention to location and shape of the lesion on anatomical images and regarding patient’s previous medical history helps to avoid interpretation pitfalls. In cholesteatoma substantially lower ADC values (range 0.5–0.9 × 10^−3^ mm^2^/s) have been reported when compared with AM (range 0.8–2.0 × 10^−3^ mm^2^/s) [18, 25, 26].

**Fig. 4:**
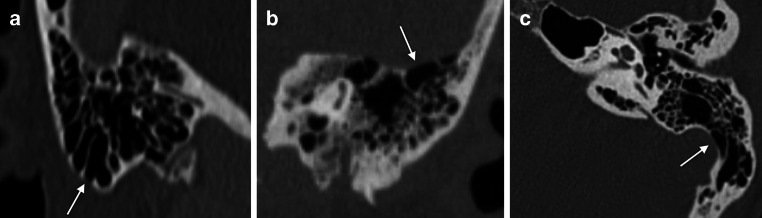
Anatomical bone pseudodehiscence (*arrows*) at the tip of the mastoid process (**a**), at the inner cortical table towards the middle cranial fossa (**b**) and the sigmoid sinus (**c**). In normally pneumatized ears, these would be regarded as anatomical variants. In case of clinical AM, they may be erroneously interpreted as inflammation-induced bone erosions

As for direct estimation of bone destruction, MRI was inferior to CT, although the results were comparable at the outer cortical table and coalescent AM as such was not missed due to multiple coexisting lesions. Estimating bone erosion is not always straightforward in CT either, as already observed from previous studies [8, 9]. Erosions develop gradually, starting as subtle demineralization and progressing contiguously to coalescence and formation of larger bone defects. Therefore, setting a definite cut-off point for bone destruction on imaging studies is challenging. The anatomical variance in shape and size of mastoid air cells and in the thickness of the compact cortical bone between individuals and at different ages compounds this challenge. For example, extension of large mastoid cells on to the surface of cortical bone may result as an anatomically pseudodehiscent cortical table (Fig. 4), which may erroneously be interpreted as pathological lesion in the context of AM.

The inner cortex especially was noticeable both for poor correlation between CT and MRI findings regarding defects and for very low interobserver agreement in MRI. Mastoid enhancement, diffusion restriction or mean ADC did not correlate with inner cortical destruction either, although they predicted bone destruction at other anatomical subsites. On T2 TSE images, internal cortical table is difficult to visualize because of juxtaposition of the sigmoid sinus with its flow-induced signal void, which renders the adjacent low-signal cortical table invisible. On T1 Gd MPRAGE images, on the other hand, the low-intensity periosteum, may simulate intact bone. It is also possible that CT overestimates bone destruction at the sigmoid sinus wall (Fig. 4c) in case of an anatomically pseudodehiscent cortical table. As we saw intracranial lesions also in patients with no inner cortical defects, it must be kept in mind that an intact inner cortical table does not rule out intracranial spread in AM.

In the future, novel MR imaging techniques may bring further advances in mastoid imaging and visualization of bony structures or their pathology. Sequences like black bone [27, 28] and ultrashort or zero echo-time (UTE and ZTE) [29, 30] or their further developments [31] with relevant postprocessing may create images with a positive bone contrast similar to CT, thereby facilitating a more intuitive interpretation by surgeons. Their true additional diagnostic potential in temporal bone MR imaging remains to be seen and may be limited by the site-specific factors such as small details and air in the pneumatized bone.

### Complications

Perisinuous lesions were detected more frequently in CT than in MRI. Discrepancies occurred in four patients with grade I and II findings. The MRI missed the linear halo (grade II) in one and small nodular halo <4 mm (grade III) in two patients, likely due to higher contrast differences in CT, which more easily depicts low-density lesions between enhancing sinus and high-density bone, whereas in MRI the adjacent bone remains signal free. Linear halo is a low-risk lesion that probably corresponds to edematous dura and does not associate with epidural abscesses [23, 32]. Previous results are discordant regarding the clinical importance of small nodular halos. A recent study showed correlation with surgically verified intracranial pathology only in grade IV lesions (nodular halo ≥4 mm thick) [32], whereas in children, epidural abscesses have also been found in association with grade III lesions [23]. The clinically most significant grade IV lesions were detected uniformly in both modalities. There were no differences between modalities in detecting epidural abscesses or venous sinus thrombosis outside the perisinuous area. Pachymeningitis and leptomeningitis, which are beyond the scope of CT [33], and one temporal lobe cerebritis were only detected in MRI. In our study, MRI also detected more extracranial complications (6 in MRI versus 4 in CT), likely due to larger imaging volume in MRI.

The results of current study did not support the hypothesis that bone destruction could be directly estimated from MRI in the same way as from CT. The inner cortical table especially remained problematic in MRI; however, based on several different MR characteristics (delineation of bony structures on T2 TSE and T1 Gd MPRAGE, intramastoid enhancement and diffusion restriction), patients could be classified into three different risk categories, according to the imaging findings (Table 3).

**Table 3:** Recommendations for further AM patient management after initial MRI

Low risk	Intermediate risk	High risk
No signs of complications AND Only mild diffusion restriction with ADC >1.2 × 10^−3^ mm^2^/s AND Only mild or moderate contrast enhancement AND No signs of osteolysis On T2 TSE or Gd T1 MPRAGE	No signs of complications BUT Suspicious or equivocal osteolysis on T2 TSE or Gd T1 MPRAGE OR Diffusion restriction with ADC ≤1.2 × 10^−3^ mm^2^/s OR Intense contrast enhancement	Intra- or extracranial complications OR Clear bone destruction on T2 TSE or Gd T1 MPRAGE
No further imaging needed, Treatment according to patient’s clinical status	Additional CT recommended	No further imaging recommended, treatment based on clinical and MRI findings

*ADC* apparent diffusion coefficient, *T2 TSE* T2 turbo spin echo, *3D T1 MPRAGE Gd* 3-dimensional isotropic T1 magnetization-prepared rapid acquisition with gradient echo with intravenous gadoterate meglumine

MRI can identify high-risk patients with extracranial or intracranial complications and clear bone lesions and these patients could be treated aggressively, based on the MR imaging findings alone. Patients without any alarming findings (no complications nor signs of bone destruction, only mild mucosal enhancement, no significant diffusion restriction with intramastoid ADC above 1.2 × 10^−3^ mm^2^/s) are very unlikely to have coalescent AM and unlikely to benefit from additional CT. Only in the third group with equivocal findings in MRI (no complications or clear bone defects, but having intense enhancement or marked diffusion restriction with ADC 1.2 × 10^−3^ mm^2^/s or less) additional CT could give valuable information on bone integrity to confirm or rule out coalescent AM.

Avoiding unnecessary CT scans in AM is important, because the majority of patients are children [16]. To date, CT has been the primary recommended modality for imaging acute mastoid infections due to its ability to detect inflammatory bone erosions, especially when modern, sub-millimeter, high-resolution temporal bone protocols are applied [9]. The CT has better availability and lower cost when compared with MRI; therefore, it is likely that CT will remain the first line imaging modality for AM also in the future. As MRI is more sensitive in detecting intracranial infections [10–15], MRI is preferred over CT in case of suspected intracranial complications. Our recommendation is to perform additional CT scans (to demonstrate bone integrity) after initial MRI, only for the intermediate risk group. Based on ALARA principles, MRI could also be the first line imaging modality for pediatric patients in situations where cost and availability do not limit its use. In young children, however, the benefits of radiation-free imaging have to be weighed against the potential hazards of anesthesia, if needed for MRI but not for CT.

With respect to contrast agents, a personalized approach must be applied when choosing the imaging modality for the patient with all the risks and benefits weighed individually. The MRI may be the preferable method in patients with iodine allergy, whereas CECT, despite the radiation hazard, is likely to possess lower fetal risk than Gd-enhanced MRI when imaging pregnant patients. Gadolinium deposition in brain tissue is a recent concern that has been detected after repeated administration of Gd-based contrast agents also in patients with normal kidney function, although firm evidence of its harmful effects is lacking according to current knowledge [34]. Both in CT and in MRI contrast agent is mainly needed for visualization of life-threatening intracranial complications (such as venous sinus thrombosis, intracranial abscesses or meningitis) and the potential benefits of its use in most cases are likely to outweigh the potential risks.

### Limitations

There are several limitations of this study. Not all patients with AM undergo radiological imaging in our institution. Therefore, our cohort probably represents patients with clinically more severe or treatment-resistant disease than average. The final number of patients enrolled was small, because only few patients had undergone both imaging studies within a maximum 48 h period (required to ensure reliable correlation between CT and MRI). The study did not include very young children (less than 2 years old), although AM incidence is the highest in this group. The main reason for this was unavailability of both CT and MRI scans in this age group. Structured surgical protocols were not available. Due to the retrospective nature of the study, there was also some variability in imaging equipment, parameters and MRI sequences used; however, this did not influence the results of the ADC ROC analysis. The material for this study has been collected over years and the MRI protocol was created in 2010 with technical possibilities available at that time. The ss EPI-DWI technique was chosen because it enabled covering the whole mastoid in a short scanning time and creating ADC maps, which was prioritized over spatial resolution or distortion-free images. By now, the DWI techniques have evolved and in our institution the sequence in AM protocol has been changed to readout-segmented echo-planar DWI (RESOLVE by Siemens Healthcare, Erlangen, Germany).

## Conclusion

The MRI did not miss coalescent mastoiditis, although it was inferior to CT in detecting bone erosions. The absence of intense enhancement and only mildly restricted diffusion (an ADC value of >1.2 × 10^−3^ mm^2^/s) had a high negative predictive value for coalescent mastoiditis. Based on the MRI findings, patients can be categorized as having low, intermediate or high risk for coalescent AM, and only the intermediate risk group is likely to benefit from additional CT.
